# Borderline decisions in brain-metastatic breast cancer: efficacy of multimodality treatments in breast cancer patients with very limited prognosis suffering from brain metastases

**DOI:** 10.1007/s10585-025-10376-9

**Published:** 2025-10-27

**Authors:** Stephanie Bendrich, Marc Alexander Wilhelm Pietrkiewicz, Markus Anton Schirmer, Hanne Elisabeth Ammon, Leif Hendrik Dröge, Laura Anna Fischer, Carla Marie Zwerenz, Charlotta Friederike Pagel-Nozari, Stefan Rieken, Julia Gallwas, Manuel Guhlich, Sandra Donath

**Affiliations:** 1 Department of Radiation Oncology, Goettingen University Medical Center (UMG), Robert- Koch-Str. 40, 37075 Goettingen, Germany; 2 Department of Gynecology and Obstetrics, Goettingen University Medical Center (UMG), Robert-Koch-Str. 40, 37075 Goettingen, Germany; 3 Comprehensive Cancer Center Göttingen (G-CCC), Goettingen University Medical Center (UMG), Robert-Koch-Straße 40, 37075 Göttingen, Germany

**Keywords:** Brain metastases, Breast cancer, WBRT, Dose escalation, GPA-score, Karnofsky performance status

## Abstract

Diagnosis of brain metastases (BM) has traditionally marked a turning point in treatment goals and prognosis of patients with breast cancer (BC). However, new systemic treatment options have both prevented the development and improved the prognosis of BC patients with BM. Stereotactic radiotherapy techniques (SRT) have widely replaced whole brain radiotherapy (WBRT) in patients with limited BM, yielding impressive survival at modest toxicity rates. However, based on established prognostic scores, many patients will not profit from modern SRT treatment concepts and, therefore, remain candidates for WBRT or best supportive care (BSC). Here, treatment decisions may be challenging. This study aims to describe clinical outcomes and explore potential prognostic factors in a single-center cohort of BC patients treated with WBRT comprising patients across the prognosis continuum reflecting everyday work routine. We retrospectively analyzed 108 patients diagnosed with BM who received WBRT between 2008 and 2020. The cohort included patients with a broad range of prognostic factors (≥ 60 years old, Karnofsky Performance Status [KPS] ≤ 70, >3 BM and a Graded Prognostic Assessment [GPA] score < 1 [anticipated life expectancy of < 4 months]), who were either treated with palliative WBRT alone or in case of focal symptoms attributed to individual location of metastasis with surgery, SRT, or WBRT + focal dose escalation (“boost”). Survival rates were estimated using the Kaplan–Meier method, and factors potentially affecting outcome were statistically assessed by the log-rank test. Uni- and multivariable Cox regression was used in survival analysis to estimate hazard ratios and to evaluate the influence of various variables on survival time. For all statistical procedures SPSS software (v. 26) was used. Median survival after diagnosis of BM was 4 months (95% CI 3–5 months). The most relevant negative prognostic factor within the group of GPA-classifiers was KPS (≤ 70), followed by the number of BM (>3). Metastasectomy displayed significantly improved survival in univariable analysis (log rank *p* = 0.008; Cox-Regression *p* = 0.018), but did not elicit as predictive for OS in multivariable analysis (*p* = 0.47). Furthermore, completion of the prescribed RT series had the strongest impact on OS in both univariable and multivariable Cox regression analyses (*p* < 0.00001) while delivery of a radiation boost in addition to WBRT exerted no significant benefit (Cox regression *p* = 0.358). Despite the continuous development of effective treatment strategies for BM, BC patients who are ineligible for innovative treatment modalities show poor survival rates. Following prognostic assessment may help to make treatment decisions. KPS and number of BM may be most important in this context. In our retrospective cohort surgical removal of BM and completion of RT series were associated with improved prognosis, while focal radiation dose escalation in addition to WBRT was not.

**Supplementary Information:**

The online version contains supplementary material available at 10.1007/s10585-025-10376-9.

## Introduction

Breast cancer (BC) is the second most common cause of brain metastases (BM), with an increasing incidence of presently 15–20% and up to 40% in autopsy cohorts [1, 2]. This notion is related to prolonged survival rates due to both improved control of primary and extracranial tumor manifestations and more frequent use of high-resolution MRI. Overall survival (OS) following the diagnosis of BM is poor and has been reported to range from 2 to 25 months [3]. Modern treatment concepts—especially in case of limited brain metastases—may yield long term survival in selected cases [4, 5] while a large proportion of patients is ineligible to profit from innovative treatment modalities. Known predictors of poor prognosis comprise number, localization, and size of BM [6].

The recursive partition analysis (RPA) by Gaspar et al. defined three prognostic classes (class 1: KPS ≥ 70, <65 years, controlled primary, no extracranial metastases; Class 2: KPS < 70, class 3: all others) based on whole brain irradiation (WBRT) as a therapeutic treatment, that allows an estimation of median survival between < 2 or > 7 months for patients with BM [7]. In 2012, an updated version of the Graded Prognostic Assessment (GPA) was published, incorporating primary tumor-specific indices. This modified GPA showed that a lower score correlates with a worse prognosis [2]. These prognostic tools were designed to support therapeutic stratification and guide decisions on whether patients might benefit from active treatment. However, evidence regarding optimal management for patients in reduced physical condition is scarce, as these patients are often excluded from clinical trials [8].

For many years, WBRT was considered to be the predominant standard of care for patients with BM but its use has been increasingly challenged due to limited efficacy and potential neurotoxicity. Prognostic classifiers such as RPA and GPA can help guide treatment decisions by allocating patients to either palliative WBRT or BSC. The randomized QUARTZ trial, a phase III non-inferiority trial in patients with BM from non-small cell lung cancer (NSCLC), demonstrated no significant differences in OS or quality of life (QoL) between BSC and WBRT in patients with KPS < 70%, supporting the selective use of WBRT in poor-performance patients [9].

In clinical routine, however, treatment decisions are often challenging, as patients within the same prognostic class can present with heterogeneous oncological features. In this study, we aimed to describe survival outcomes and explore prognostic factors in a large, monocentric, real-world cohort of BC patients receiving WBRT. While most patients had poor general condition and low GPA scores, the cohort also included patients across the prognosis spectrum, reflecting everyday clinical practice. By examining the impact of individual prognostic indicators, we sought to provide insights to the potential benefits of WBRT and support clinical decision-making for patients with limited life expectancy.

## Patients and methods

### Patient population

**Fig. 1:**
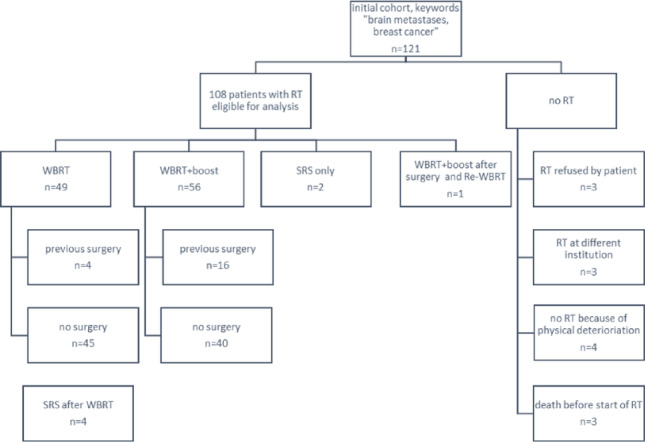
Patient selection for database

In this single-center retrospective study, 108 BC patients with BM who had received BM-directed radiotherapy (RT) at the Department of Radiation Oncology, University Medical Center, Goettingen between 2008 and 2020, were included (Fig. 1). The study was approved by the Institutional Ethics Committee (confirmed on 05/17/24). The data collected included age at initial diagnosis (ID) and at diagnosis of brain metastases (DBM), respectively, time between ID and DBM, KPS at initial diagnosis of BM, number of BM, localization of BM, tumor stage and tumor characteristics (histopathology and immunohistochemistry [IHC]), systemic tumor burden, type of systemic therapy, type of local treatment of BM, duration and dosage of irradiation of BM treatment, as well as date of death or last contact. Synchronous BM were defined as occurrence of BM within 6 months after ID, metachronous BM as the occurrence of BM > 6 months after ID. The GPA Score was calculated as previously published [2] at time of data collection (Table 1). While the majority of patients were ≥ 60 years old, had a KPS ≤ 70, > 3 BM and a GPA score < 1 (Tables 2, 3, 4)—indicating a poor prognosis with a mean life expectancy of < 6 months—the cohort also included patients with more favorable prognostic parameters, reflecting real-world clinical practice.

**Table 1:** Graded prognostic assessment (GPA); Gaspar et al. 7; extracranial metastases here represent a dichotomized feature and thus a score of either “0” or “1” is assigned

	Score		
	0	0,5	1
Age	> 60	50–59	< 50
Karnofsky performance status	< 70	70–80	90–100
Number of BM	> 3	2–3	1
Extracranial metastases	Present	–	none

**Table 2:** Calculated GPA-score

GPA-score	All patients (*n* = 108)	
	*n*	%
0–1	57	52.78
1.5–2.5	45	41.67
3	4	3.70
3.5-4	2	1.85

**Table 3:** Systemic and cerebral metastatic burden *synchronous defined as metastases at ID or within 6 months after ID; metachronous defined as > 6 months after ID **no HR-status was obtained for one patient ***no Her2neu-status was obtained for 5 patients

Systemic metastatic burden	All patients (*n* = 108)	
	*n*	%
Synchronous* extracranial metastases	**22**	20.37
TNBC	6	5.56
Her2neu +	8	8.33
other	8*	6.48
Metachronous* extracranial metastases	**86**	79.63
TNBC	20	18.52
HER2neu +	18	16.67
Other	48***	46.29
Cerebral metastasic burden		
Synchronous* BM	**7**	6.48
TNBC	2	1.85
HER2neu +	4	3.70
Other	1	2.78
Metachronous* BM	**101**	93.52
TNBC	24	23.15
HER2neu +	24	22.22
other	53	48.15
Number of metastases		
Single cerebr. Met	13	12.04
2–3 cerebr. Met.	16	14.81
> 3 cerebr. Met.	79	73.15
Meningiosis carcinomatosa	15	13.89
Site of involvement		
Supratentorial involvement only	37	34.26
Infratentorial involvement only	6	5.56
Supra- and infratent. involvement	65	60.19

**Table 4:** Patient and tumor characteristics *Missing values to 108: no information was documented

Patient and tumor characteristics	All patients (*n* = 108)	
	*n*	%
Age at diagnosis of BM		
≤ 50	33	30.56
51–59	30	27.78
≥ 60	45	41.67
Karnofsky performance status		
≤ 50	18	16.67
60–70	52	48.15
80–100	38	35.19
Histopathology		
NST	87	80.55
G3	40	45.97
Other histopathology	21	19.44
G3	8	38.09
Immunohistochemistry of primary*		
Her2neu+/HR+	13	12.04
Her2neu+/HR -	15	13.89
Her2neu-/HR+	45	41.67
TNBC	26	24.07
Immunohistochemistry shift in BM		
HR+ -> HR -	3	14.29
HER2neu- -> Her2neu+	2	9.52
Neurological symptoms		
Global symptoms	26	24.07
Focal symptoms	80	74.07
No symptoms	2	1.85
Edema		
Yes	87	80.55
No	21	19.44

### Patient treatment

Following multidisciplinary tumor conferences, all patients were thoroughly informed about their limited prognoses and the possibility of BSC. Especially in case of additional extensive extracerebral tumor burden, systemic treatment was continued during RT. All patients included in this study received WBRT. In the case of focal symptoms attributed to individual BM localizations, or in cases with increased intracerebral pressure from large BM, surgery, SRT, or additional dose delivery (“boost”) was performed in addition to WBRT. A fully applied RT dose was defined as > 80% of the prescribed dose, ensuring treatment adequacy.

### Statistical analysis

Overall survival (OS) rate was defined as the primary endpoint calculated from the date BM was diagnosed to death. Patient-, tumor-, and treatment-derived parameters were tested for impact on OS. Therefore, the Kaplan–Meier method was employed, and statistical assessment was carried out using the log-rank test. Univariable Cox regression analysis was performed for evaluation of hazard ratios of single items regarding the primary endpoint. P values < 0.05 were considered as statistically significant in an exploratory fashion without adjusting for multiple comparisons. Variables showing an association with the primary endpoint at *p* < 0.1 in the univariable analysis were included in a multivariabe Cox regression model. A stepwise approach was applied to identify the strongest associations and to eliminate potentially intercorrelated variables, whereby preventing overfitting of the model. The final model comprises variables with associations at *p* < 0.05. Statistical procedures were executed by use of SPSS software (v26, IBM, Chicago, IL, USA).

## Results

### Patients and tumor characteristics

**Fig. 2:**
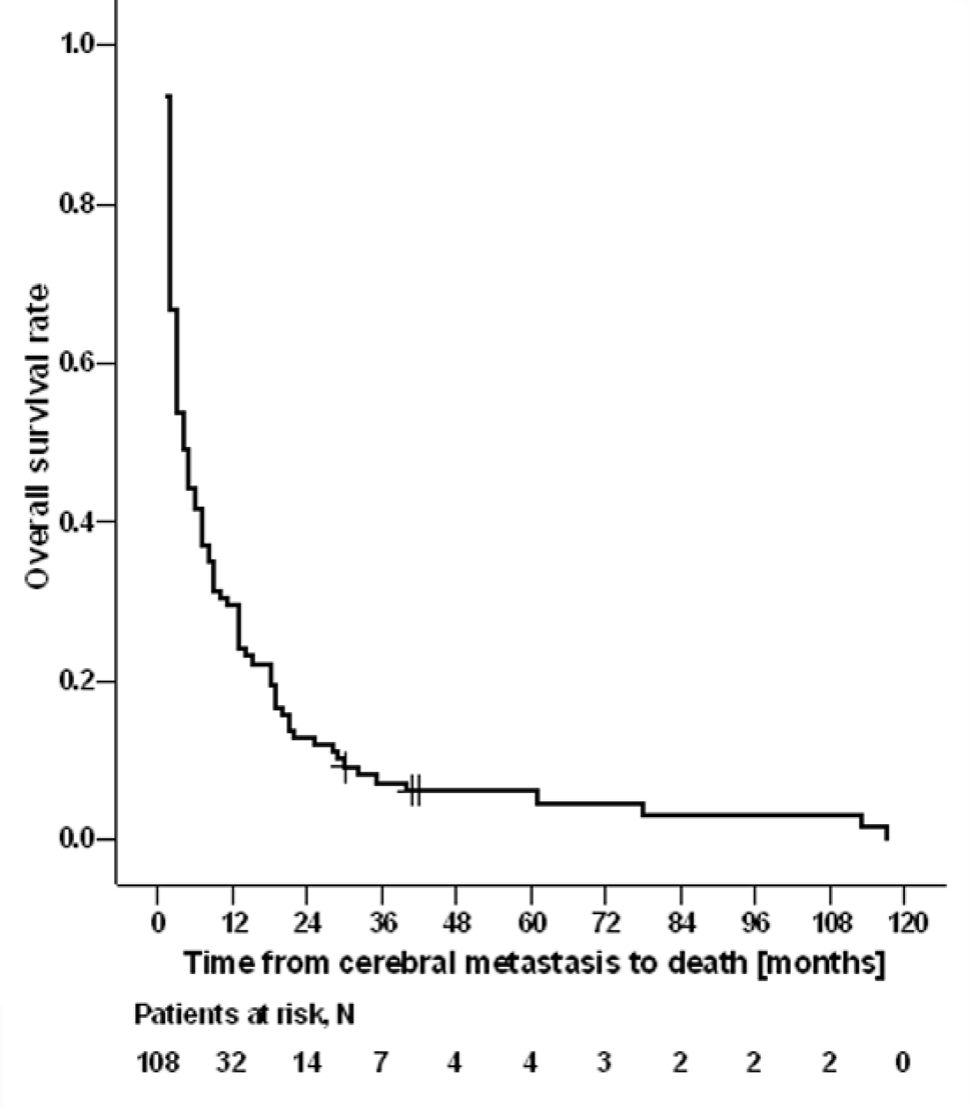
OS rates after DBM

**Fig. 3:**
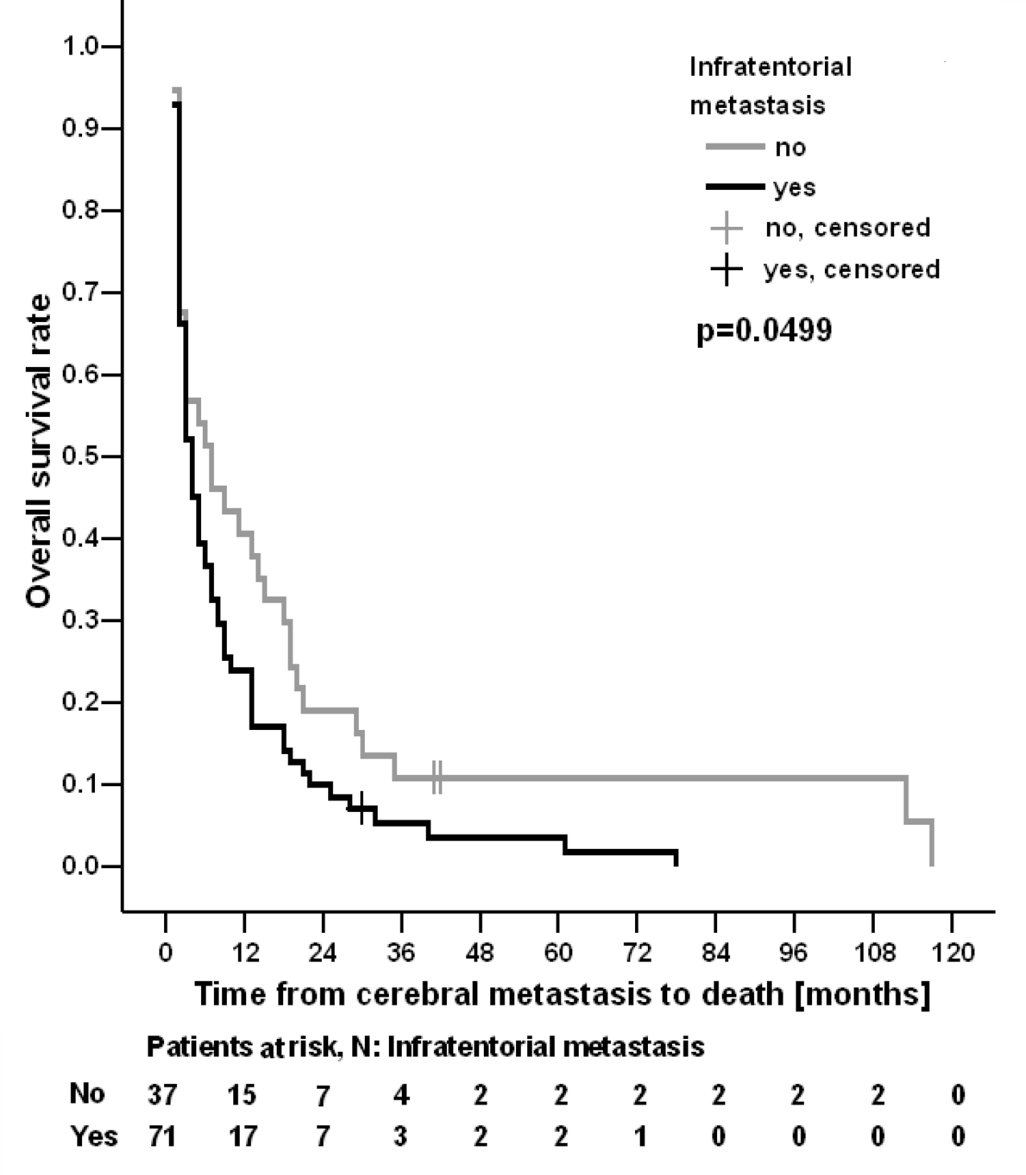
OS stratified by localisation of BM

Between 2008 and 2020, 108 patients received RT for BM. Median time between ID and detection of BM was 48 months (95% CI 35–61 months). The mean age at ID and at DBM was 51.6 years (range 32–81) and 58.1 years (34–86), respectively. Median time from ID to death or last follow-up was 65 months (95% CI 46–84 months), and median OS was 4 months (95% CI 3–5 months) (Fig. 2). In seven patients (6%), synchronous cerebral metastatic breast cancer (as defined above) was present; in 101 (94%) metachronous cerebral metastatic breast cancer was diagnosed. 22 patients had distant extracerebral metastases at the time of initial diagnosis, 86 patients were diagnosed with distant extracerebral metastases during the course of the disease (Table 1). Concerning tumor types, no difference in the presence of metachronous or synchronous extracerebral metastases was observed, neither in a triple negative breast cancer (TNBC) nor a Her2-new positive tumor. However, with regard to cerebral metastatic burden, singular BM were significantly more frequently associated with TNBC (*p* = 0.009). Concerning cerebral metastatic burden, the metastatic pattern varied from singular metastasis to disseminated intracerebral metastases with > 100 metastases (Table 3; Fig. 7, suppl). Strictly infratentorial involvement was associated with significantly reduced OS from DBM until death in the log-rank test (*p* = 0.0499, Fig. 3). A very similar association was observed in the univariable Cox regression analysis, with a hazard ratio of 1.478 (95% CI, 0.972–2.246). The lower bound of the 95% confidence interval was slightly below 1.0, and thus the corresponding p-value was marginally higher than that in the log-rank test, while still indicating a statistical trend (*p* = 0.068; Table 7, supplement). The majority of patients had focal neurological deficits (Table 2). Leptomeningeosis was present in 15 patients (14%).

**Fig. 4:**
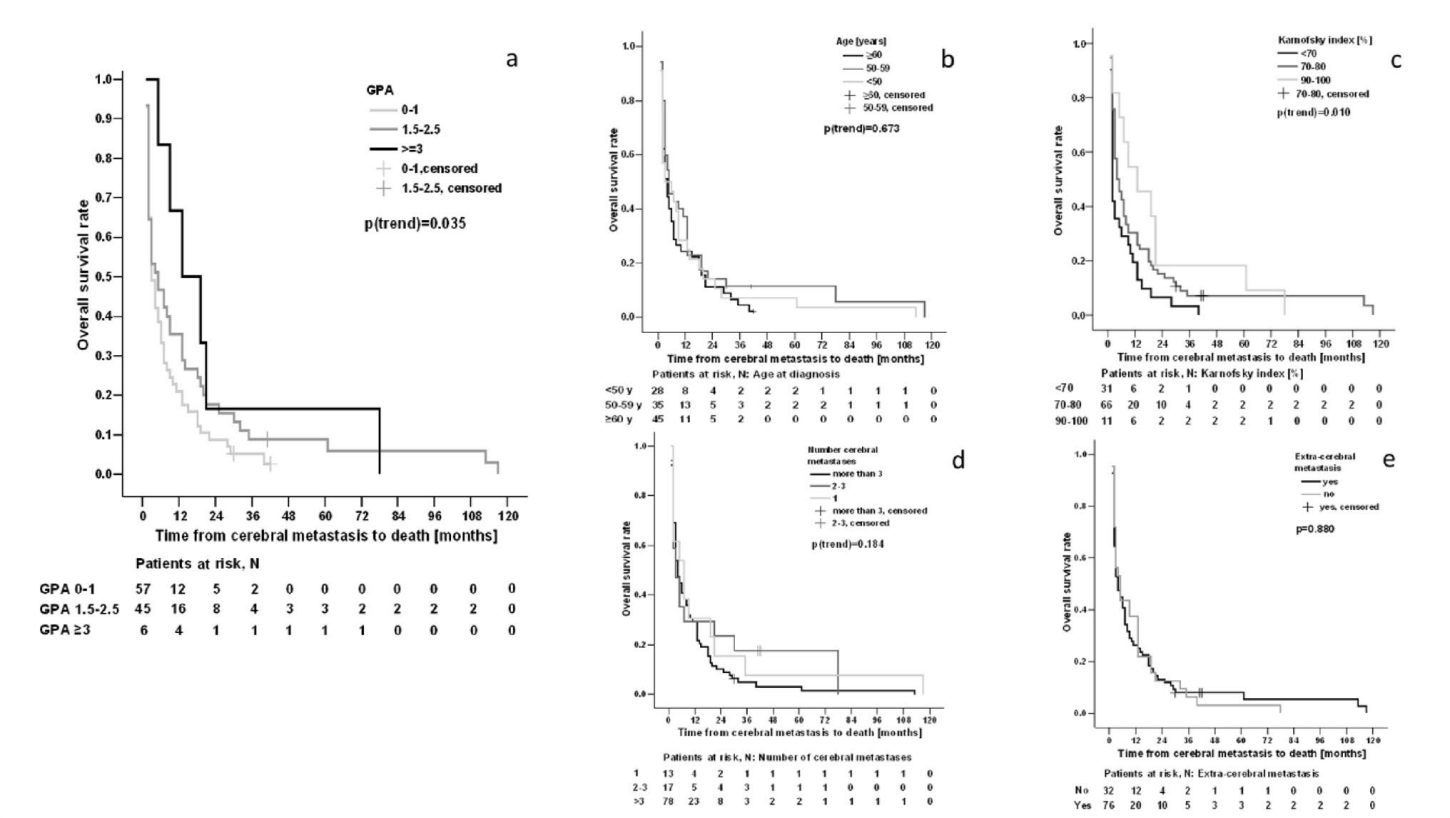
Cumulative OS rates for GPA (**a**) and itemized for single GPA classifiers (**b**–**e**)

In the subsequent calculation of the GPA score, median survival times for the different groups depicted as follows: GPA 0–1: 3 months (95% CI 2–4 months), GPA 1.5–2.5: 5 months (95% CI 2–8 months), GPA ≥ 3: 13 months (95% CI 1–25 months) (Fig. 4). We examined the individual impact of each classifier within the GPA-Score, whereby KPS (≤ 70) proved to be the most relevant influencing variable in relation to OS (Fig. 4c; Table 7 supplement) and age as well as extracranial tumor burden and number of BM (> 3) were not. The negative impact of a KPS ≤ 70% was also proven in the multivariable analysis (HR 2.00; 95% CI 1.28–3.13, *p* = 0.002, Table 8, supplement).

### Treatment characteristics

**Fig. 5:**
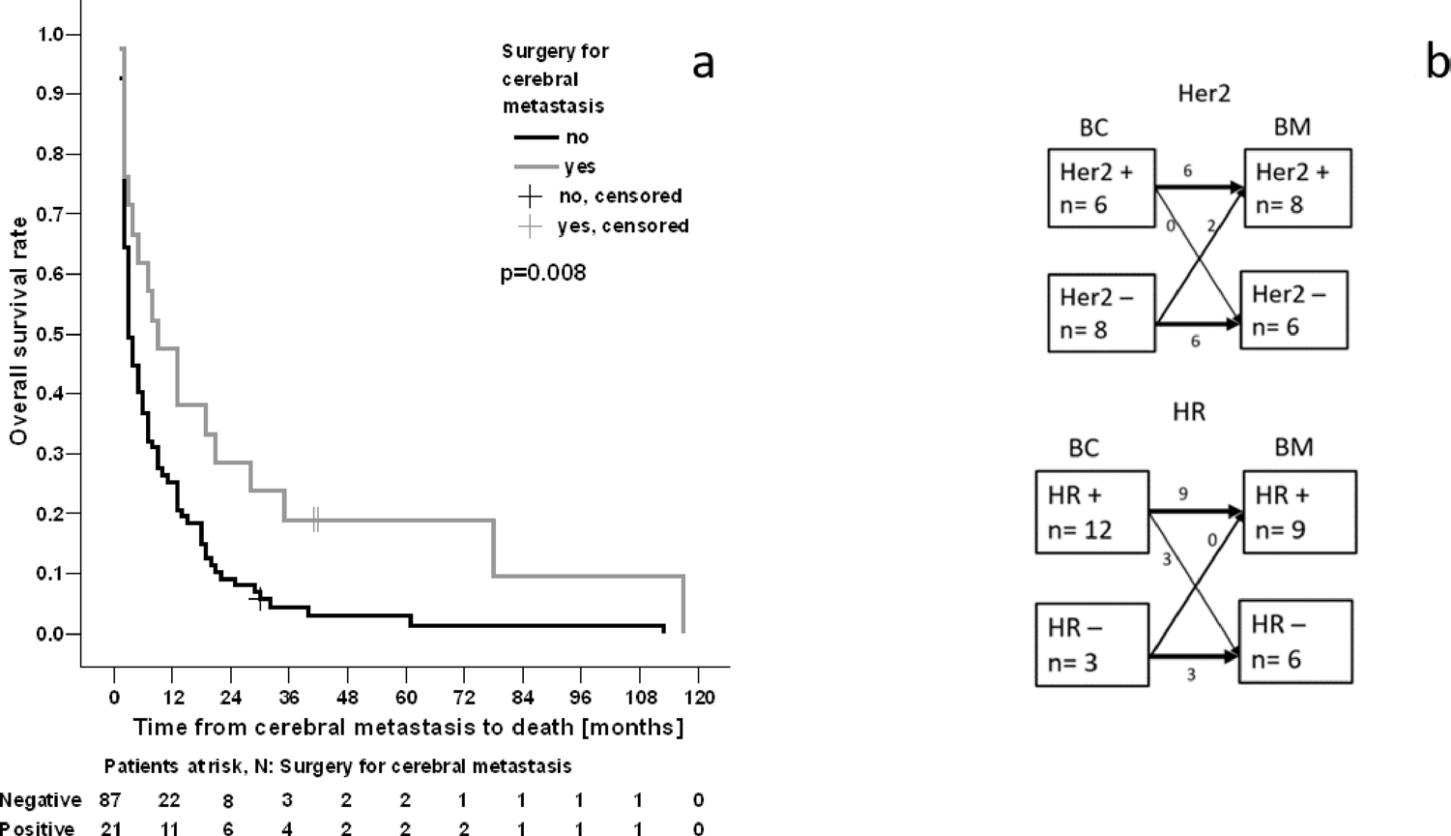
OS rates for metastasectomy followed by WBRT +/- boost (**a**); Receptor conversion for HR and Her2 (**b**) (21 patients underwent surgery, 6 TNBC without receptor conversion, 14 left for analysis (dropout of 1 patient because of missing postoperative histopathology; one patient with bilateral BC (*n* = 15 for HR)

For local treatment, 21 patients (19%) were diagnosed with symptoms attributable to the localization of BM and underwent metastasectomy, of whom 17 (16%) subsequently received WBRT + boost radiation of the resection cavity and 4 patients WBRT only. As further focal treatments, 2 patients received definite SRS only with doses varying from 13 to 18 Gy dosed at 80% isodose, 4 patients were treated with SRS after a previous WBRT with a dosage varying from 13 to 24 Gy dosed at 50–80% isodose. All other patients received WBRT (*n* = 46; 42.59%) or WBRT + boost administration (*n* = 40; 37.04%, Fig. 1). Techniques used were restricted to 3D-conformal RT alone or in combination with volumetric modulated arc therapy (VMAT) or intensitiy-modulated RT (IMRT)-techniques in the case of a boost volume or VMAT alone (Fig. 8 suppl. and Table 5). Patients that underwent metastasectomy had significantly improved survival compared to the non-metastasectomy group (log rank *p* = 0.008, Fig. 5a) as well as in univariable cox-regression (*p* = 0.014, hazard ratio of 0.52 with 95% CI 0.31–0.88, Table 7 supplement). However, in multivariable analysis, metastasectomy did not elicit as predictive for OS (*p* = 0.40, removed during stepwise iterations and thus not contained in Table 8, supplement).

**Table 5:** Local and systemic treatment (RT = radiotherapy, wbrt = whole brain radiotherapy, srs = stereotactic radiosurgery, vmat = volumetric modulated Arc radiotherapy, imrt = intensity modulated Radiotherapy)

Local treatment	All patients (*n* = 108)	
	*n*	%
OP	21	19.44
RT interruption (2-11d)	6	5.56
RT complete	88	81.48
WBRT only	49	45.37
WBRT + boost	56	51.85
WBRT + boost followed by WBRT	1	0.926
SRS		
As definite treatment (13–18 Gy@80% isodose)	2	1.85
After previous WBRT (13–24 Gy@50–80% isodose)	4	3.70
Technique WBRT		
3D-conformal RT	56	51.85
VMAT	49	45.37
IMRT	1	0.93
Boost technique		
Multi-isocentric fields	5	4.63
VMAT	52	48.15
Systemic treatment		
Antihormonal therapy	53	49.07
Trastuzumab	31	28.70
Pertuzumab	7	6.481
CDK4/6 Inhibitor	1	0.926
Chemotherapy	94	87.04
Bisphosphonate	39	36.11
Dexamethason	91	84.26

For WBRT, the distribution of patients receiving a boost versus no boost was well balanced between the groups. Administration of a boost did not significantly influence OS from the time of DBM to death (*p* = 0.83, Table 7 supplement). When stratifying patients by GPA, no significant survival differences were observed between those receiving a boost versus no boost (GPA 0–1: *p* = 0.3; GPA 2-2.5: *p* = 0.5; GPA ≥ 3: number of cases was too small to allow a valid conclusion) (Table 6). In contrast, receiving an almost complete RT series (defined as either 80% of the prescribed irradiation dose or ≥ 30 Gy) was favorable and had the strongest impact on OS in both univariable and multivariable Cox regression analyses (*p* < 0.00001; Tables 7 and 8 supplement). Further variables with an impact of *p* < 0.05 in this multivariable model comprised the above-mentioned KPS ≤ 70%, which reflects poor general health, as well as infratentorial metastasis and application of chemotherapy—all three of which were associated with worse outcome.

**Table 6:** GPA subgroup analysis for WBRT+/- boost (*only 6 patients, 5 with boost, one without)

GPA-Score	*p*-value
0–1	0.3
1.5–2.5	0.5
≥ 3	0.025*

Comparing IHC status between ID and DBM, all patients with preoperatively TNBC remained triple negative; in 3 patients a shift from HR positivity to negativity was documented (Fig. 5b). A positive effect on survival from ID to death was seen for HR-positive patients, with significant influence of ER (*p* = 0.03; HR 0.63 with 95% CI 0.41–0.9) and nearly significant influence of PR (*p* = 0.06; HR 0.67 with 95% CI 0.44–1.02). Her2neu receptor positivity had no influence neither on survival from ID to death nor on OS. There was no difference in OS comparing the TNBC group to non-TNBC patients (*p* = 0.50 log-rank).

**Fig. 6:**
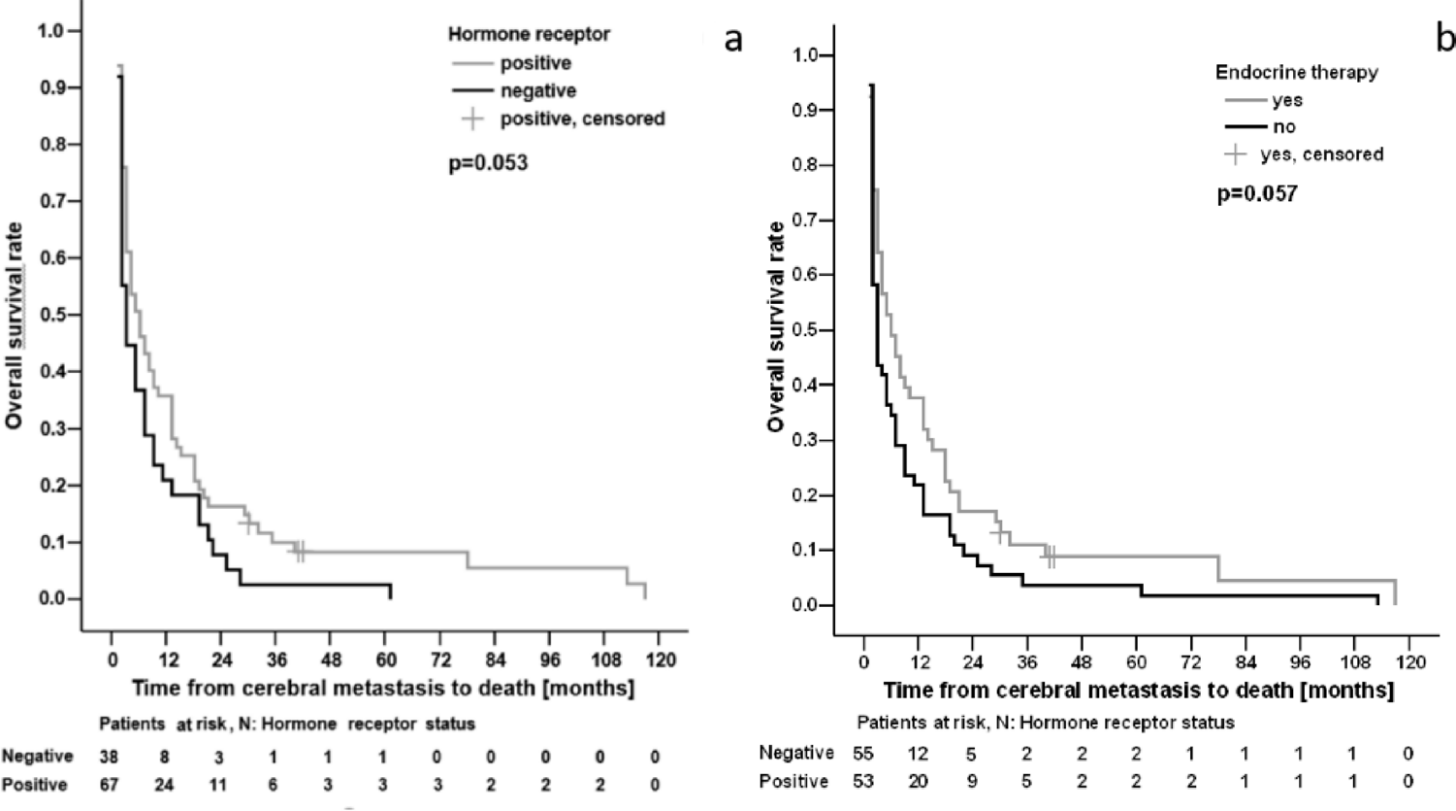
Effect of HR-status (**a**) and endocrine therapy (**b**) on OS

Concerning systemic therapies, only antihormonal therapy was associated with significantly improved survival from ID to death (*p* = 0.00002; HR 0.41 with 95% CI 0.27–0.62) but not from DBM to death (Fig. 6).

No grade 3 acute toxicity or higher was reported in relation to RT. Due to short survival time, no analysis of neurocognition was possible.

## Discussion

In this study, we retrospectively analyzed the outcome of local treatments in a cohort of BC patients diagnosed with BM and a poor prognosis as suggested by the GPA score [7]. Our cohort consisted predominantly of patients with a poor prognosis due to high age (41.67% 60 years), poor clinical performance (64.81% with a KPS < 70%), high extracerebral tumor burden, a large proportion of poorly differentiated primary tumors (G3), and the high number of BM (73.15% with > 3). According to GPA, a mean life expectancy of ≤ 6 months was anticipated after WBRT. While GPA provides a reliable prognostic estimate, we found that individual classifiers such as KPS and number of metastases may be more informative for guiding treatment decisions in clinical practice. Our cohort reflects real world patients who are typically excluded from clinical trials due to poor performance status or extensive disease. Decisions regarding therapy versus best supportive care (BSC) remain ethically challenging, emphasizing the need for individualized assessment beyond composite scores.

Furthermore, there is an absence of particular knowledge about the management of patients with BM in BC, as the majority of studies are not BC-specific but frequently encompass several entities or attempt to draw analogies from different tumor types, e.g. NSCLC. As the most aggressive subtype with the worst prognosis of BC, TNBC accounts for about 15–20% of all BC, of which approx. 5% of patients present with metastatic disease at ID [10]. At the time of TNBC diagnosis, the incidence of de novo brain metastases is approximately 14%, markedly above that observed in HR-positive and HER2-positive breast cancer [11]. Our cohort comprised slightly higher, although still comparable rates for TNBC (24.07%, Table 4) among all BC subtypes. In our cohort, there were 23.08% of TNBC cases with extracranial and 7.69% with BM metastasis in de novo-diagnosed disease. Consequently, our cohort also reflects the reality in the treatment of patients with BM in BC outside of clinical trials, since the latter predominantly include patients in very good general condition and usually with addressable tumor targets [11].

Beyond the GPA score, the quantity of metastases, along with their location, is frequently identified as a prognostic factor, with infratentorial location linked to a worse prognosis due to its association with critical neighboring structures (brainstem, cranial nerves) and the resultant therapeutic difficulties [12]. This association remains unproven but plays an important role in clinical routine for treatment decisions [12]. In our cohort, we were able to demonstrate a significantly poorer OS for patients with infratentorial or combined supra-/ and infratentorial metastases (log rank *p* = 0.049 and *p* = 0.02, respectively), warranting consideration in therapeutic decision-making. Moreover, we confirmed that local treatment via surgery was associated with a significantly improved OS compared to patients who received WBRT alone or in combination with a boost (*p* = 0.008). These findings are supported by the literature [13], where patients who received local (ablative) therapy for their BM had a prolonged brain metastases-free survival and better in-brain control [14, 15]. Our data now extend these reports, demonstrating also a benefit for resection in terms of OS. Surgery can lead to improved OS through better symptom management (reduction of intracranial pressure) and thus enhanced quality of life. In addition, surgery provides valuable further information regarding IHC, enabling the reassessment of systemic treatment options with targeted therapies (see below). Due to the small number of patients in the subgroup, it is not possible to draw any conclusions about the effectiveness of SRS or SFRT.

In 1997 the RTOG initiated evaluations of treatment efficacy, namely WBRT, for patients with BM. Since then, prognostic scores have been developed, including the GPA and the disease-specific GPA (DS-GPA) as a recent update. These scores represent a valid instrument for categorizing BM patients based on median survival rates and determining a treatment strategy. The median OS times of our patients, stratified by the GPA score, align with the results reported by Sperduto et al. [2]. In our cohort, treatment decisions were mostly based on the KPS rather than the GPA score, resulting in patients who may no longer qualify for therapy according to the subsequently calculated GPA score but receiving treatment in the form of WBRT. The median survival time for the GPA 0–1 group in our cohort was 3 months, with the KPS identified as the most relevant prognostic classifier. It thus can be argued that patients with a low GPA score resulting from a high extracranial metastatic burden, a high(er) number of cerebral metastases or age, may still be treated based on an acceptable KPS score, as even a few months of survival can make a decisive difference for those affected, particularly in highly palliative situations.

WBRT has been proven to lead to substantial deterioration of neurocognition [16, 17, 18, 19], is increasingly questioned, and should be reserved for patients with multiple brain metastases, limited prognosis and reduced general condition or as a salvage treatment option in lieu of best supportive care (BSC). Especially in cases of patients with asymptomatic BM and poor prognosis, data from the QUARTZ-trial suggest that WBRT does not provide any benefit compared to BSC [9]. Recent data demonstrate, that sparing the hippocampal area during radiotherapy leads to less neurocognitive deterioration and should be considered in patients with a favorable prognosis [18]. Derived from studies supporting the use of dose-escalated SRS [16, 13, 20, 21, 22], several trials have investigated the potential benefit of focal radiation boosts delivered in addition to WBRT to promote superior local control or even survival [17, 19, 23]. In our cohort, 51.85% received radiation boosts; however, we were not able to identify any clinical benefit of these focal dose escalations. The only significant influence on OS after DBM was the completion of irradiation (*p* < 0.00001). Of all the classifiers of the GPA score, the KPS is probably best suited to assess whether a patient is likely to complete irradiation completely and without interruptions and will therefore benefit from the intended therapy. Concerning neurotoxicity, no acute grade 3 toxicity or higher was reported under irradiation therapy. Late-onset neurotoxicity was not documented and therefore could not be reported. However, we were able to show that additional surgery was associated with a significantly improved OS (*p* = 0.008) compared to WBRT (+/- boost) alone.

The receptor status is crucial for therapeutic decision-making especially for patients with highly aggressive tumor biologies like TNBC, HER2neu + or HR-negative BC. It significantly influences the efficacy of systemic treatments. Achieving cerebral control remains challenging despite advancements in new therapeutic options. Historically, systemic therapy for metastatic breast cancer was frequently determined by the receptor status of the initial tumor. Recent studies indicate a discrepancy between the receptor profiles of initial BC and later metastases. This receptor conversion plays an important role in the management of metastatic BC as it may lead to inappropriate endocrine or Her2neu-targeted therapy and especially Her2neu receptor conversion may serve as an independent prognostic factor on OS [20]. Mainly in the case of BM, administration of (targeted) systemic therapies is associated with a better prognosis [22]. Consequently, bioptic verification of disease progression or metastases and re-evaluation of IHC are strongly advised to adequately address any tumor burden and prevent inappropriate therapies. Our cohort demonstrated receptor conversion amongst patients who received surgery in 23.81% (14.29% for HR and 9.52%Her2neu respectively) (Fig. 5b). Conversion rates of 29–50% for HR and 11% for Her2neu receptors are reported [24], but are limited to small case series and provide conflicting results regarding effects on OS [20]. In our cohort, only administration of AHT had a significant impact on OS from time of ID (*p* < 0.0001), but it showed only a trend-like association on survival from DBM to death (*p* = 0.057, Fig. 6b). This suggests that the otherwise systemically effective AHT fails to achieve adequate concentrations in the brain, as the blood-brain barrier represents a substantial barrier [25].

Alongside Ribociclib, Abemaciclib was a promising active substance in the treatment of HR+, HER2– metastatic BC in combination with AHT [26]. Despite cerebral accumulation, there was no increased efficacy in patients with BM, indicating that still no substance with increased intracerebral activity is available for this group [27, 28].

The majority of patients suffering from BM have a Her2neu + IHC. Two studies have investigated the influence of an additional tyrosine kinase inhibitor (TKI) or Trastuzumab-Deruxtecan (T-Dxd) with regard to freedom from progression and survival [29, 30]. Both were able to achieve a significant improvement in this cohort, meaning that two systemic treatment options are available for patients with Her2neu-positive BC to achieve disease control beyond local interventions - such as surgery or RT - or to postpone WBRT in order to prevent neurocognitive deficits and decline in QoL.

Finally, several limitations of the present study must be acknowledged. These include its retrospective design and associated issues such as partly incomplete data (e.g., receptor status, information on systemic treatments), small sample sizes in subgroup analyses (e.g., the SRS group), as well as the heterogeneity and imbalance of the patient population, which may have introduced bias. Furthermore, WBRT is no longer standard of care, as previously mentioned, but local therapy is recommended as long as it is reasonable, feasible, and compatible with the tumor burden, general condition and prognosis of the patient. Nevertheless, we hereby provide an analysis of real-world data of a cohort with poor to very limited prognosis and thus instances from everyday clinical practice in the complex care of highly palliative patients.

## Conclusion

In this retrospective cohort of breast cancer patients with brain metastases and predominantly poor prognosis, WBRT was feasible and could be completed in most patients. While our data suggest that selected patients with an acceptable KPS may derive some benefit from WBRT, the overall prognosis remains limited. Therapeutic decision-making is complex and should consider multiple factors, including performance status, number and location of metastases and patient preferences. The GPA score can serve as a helpful tool for prognostic stratification, but individual classifiers — particularly KPS — may provide more relevant guidance for treatment selection than the composite score alone. These findings emphasize that WBRT should be considered on a case-by-case basis in patients with low GPA scores, rather than being applied solely on the basis of score thresholds.

## Supplementary Information

Below is the link to the electronic supplementary material.

Supplementary Material 1

Supplementary Material 2

Supplementary Material 3

Supplementary Material 4

## Data Availability

No datasets were generated or analysed during the current study.
