# Supplementary figures and images for: Borderline decisions in brain-metastatic breast cancer: efficacy of multimodality treatments in breast cancer patients with very limited prognosis suffering from brain metastases
Source: Clin Exp Metastasis. 2025 Oct 27;42(6):62. doi: 10.1007/s10585-025-10376-9 (PMC12559089; doi:10.1007/s10585-025-10376-9)

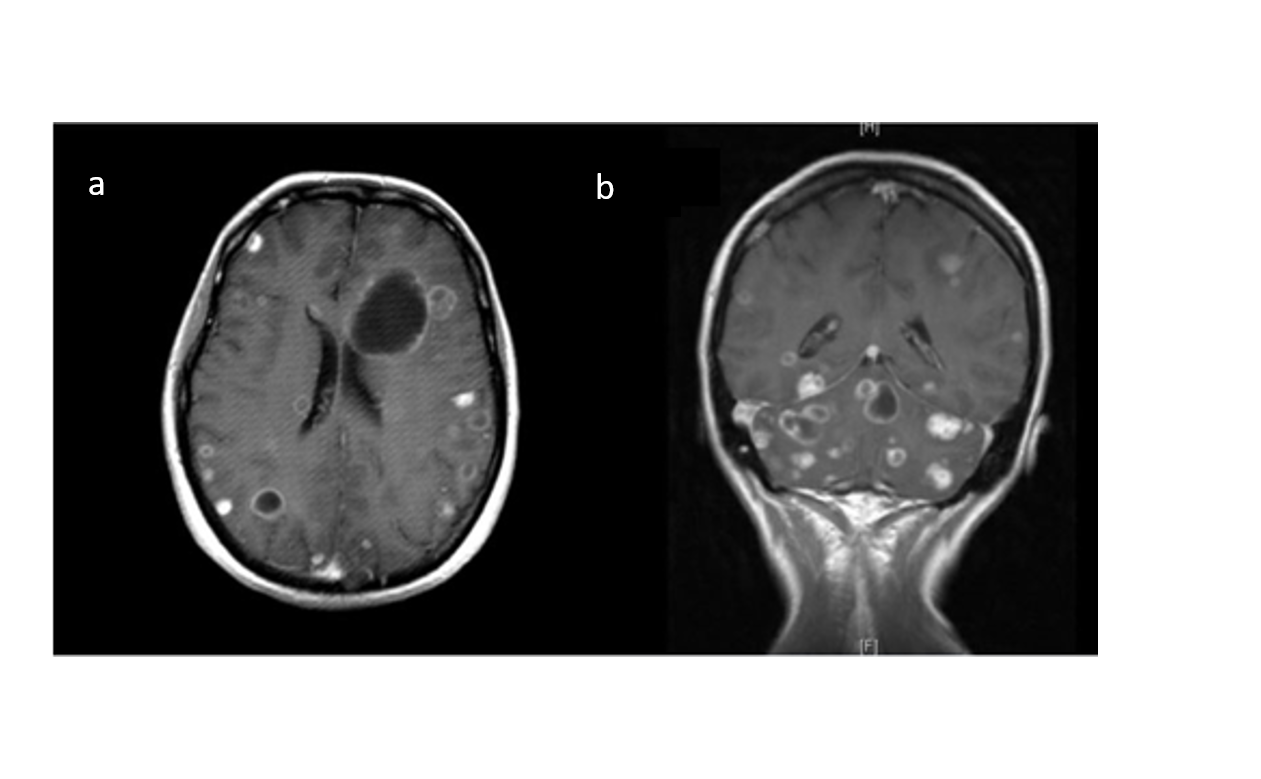

Supplement: Supplementary file 1 — Supplementary Material 1 [file 10585_2025_10376_MOESM1_ESM.tif]

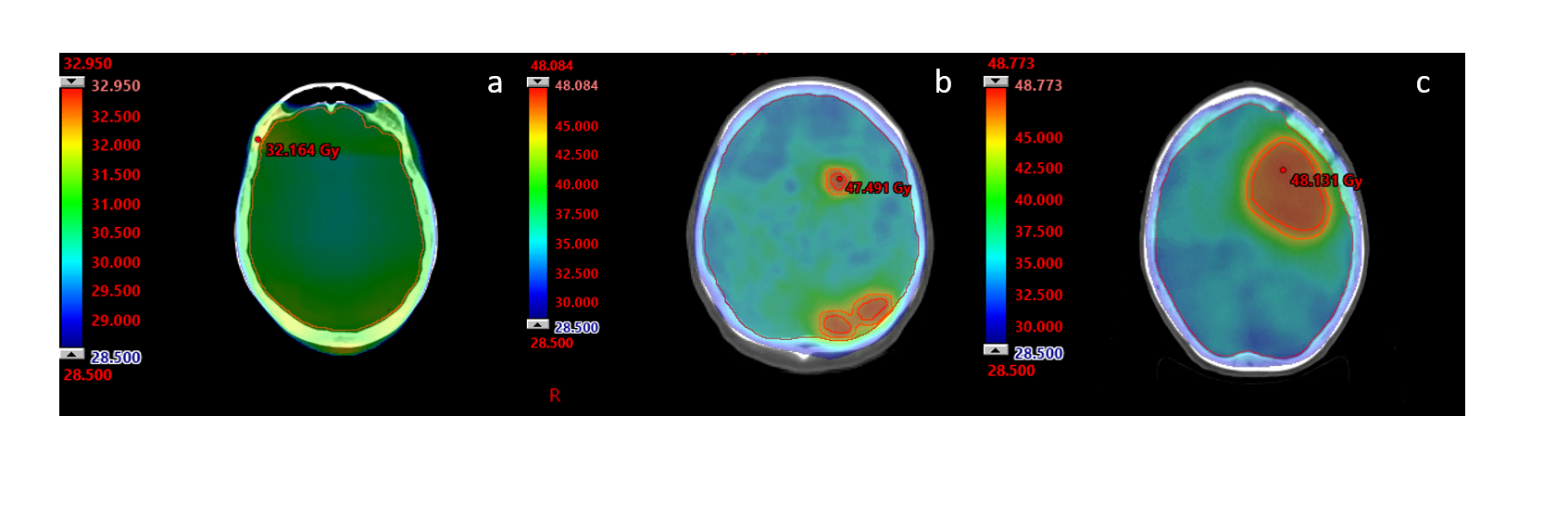

Supplement: Supplementary file 2 — Supplementary Material 2 [file 10585_2025_10376_MOESM2_ESM.tif]
