# Supplementary material for: Borderline decisions in brain-metastatic breast cancer: efficacy of multimodality treatments in breast cancer patients with very limited prognosis suffering from brain metastases
Source: Clin Exp Metastasis. 2025 Oct 27;42(6):62. doi: 10.1007/s10585-025-10376-9 (PMC12559089; doi:10.1007/s10585-025-10376-9)
Supplement: Supplementary file 3 — Supplementary Material 3 [file 10585_2025_10376_MOESM3_ESM.docx]

| **Variable** | **Category level**  **(No. of patients)** | **Hazard ratio (HR)** | **95% CI für HR** | **P value (Cox)** |
| --- | --- | --- | --- | --- |
| GPA score | **1.5-3.5 (51) vs. 0-1 (57)** | **0.696** | **0.468 – 1.035** | **0.074** |
|  | 1-3.5 (78) vs. 0-0.5 (30) | 0.691 | 0.447 – 1.070 | 0.097 |
|  | 2.5-3.5 (15) vs. 0-2 (93) | 0.850 | 0.491 – 1.472 | 0.563 |
|  | 3-3.5 (6) vs. 0-2.5 (102) | 0.583 | 0.254 – 1.337 | 0.203 |
| Karnofsky Performance Status | ≤90% (104) vs. 100% (4) | 1.653 | 0.604 – 4.522 | 0.328 |
|  | ≤80% (97) vs. ≥90% (11) | 1.542 | 0.820 – 2.901 | 0.179 |
|  | **≤70% (70) vs. ≥80% (38)** | **2.087** | **1.347 – 3.232** | **0.001** |
|  | ≤60% (31) vs. ≥70% (77) | 1.662 | 1.084 – 2.550 | 0.020 |
|  | ≤50% (18) vs. ≥60% (90) | 1.497 | 0.897 – 2.499 | 0.122 |
|  | ≤40% (18) vs. ≥50% (90) | 1.367 | 0.688 – 2.717 | 0.373 |
|  | ≤30% (6) vs. ≥40% (102) | 1.470 | 0.639 – 3.382 | 0.365 |
| No. cerebral metastases | ≥2 (95) vs. 1 (13) | 1.307 | 0.713 – 2.398 | 0.387 |
|  | ≥3 (86) vs. 1-2 (22) | 1.389 | 0.846 – 2.280 | 0.194 |
|  | ≥4 (78) vs. 1-3 (30) | 1.347 | 0.862 – 2.103 | 0.191 |
|  | ≥5 (73) vs. 1-4 (35) | 1.213 | 0.796 – 1.849 | 0.369 |
|  | ≥6 (71) vs. 1-5 (37) | 1.131 | 0.747 – 1.713 | 0.560 |
|  | ≥7 (69) vs. 1-6 (39) | 1.090 | 0.725 – 1.640 | 0.678 |
|  | ≥8 (65) vs. 1-7 (43) | 1.015 | 0.681 – 1.513 | 0.940 |
|  | ≥9 (62) vs. 1-8 (46) | 1.089 | 0.735 – 1.615 | 0.669 |
|  | ≥10 (61) vs. 1-9 (47) | 1.085 | 0.733 – 1.606 | 0.683 |
|  | ≥11 (60) vs. 1-10 (48) | 1.098 | 0.743 – 1.622 | 0.639 |
|  | ≥21 (51) vs. 1-20 (57) | 1.015 | 0.690 – 1.494 | 0.939 |
| Age | Per year | 1.006 | 0.988 – 1.023 | 0.524 |
| Grading^1^ | G3 (48) vs. G2 (55) | 1.233 | 0.826 – 1.839 | 0.306 |
| Supratentorial metastasis | Yes (102) vs. No (6) | 1.151 | 0.500 – 2.646 | 0.741 |
| **Infratentorial metastasis** | **Yes (71) vs. No (37)** | **1.478** | **0.972 – 2.246** | **0.068** |
| Metastases with edema | Yes (86) vs. No. (22) | 0.970 | 0.594 – 1.586 | 0.904 |
| **Surgery for cerebral metastasis** | **Yes (21) vs. No (87)** | **0.521** | **0.310 – 0.876** | **0.014** |
| Meningeosis carcinomatosa^1^ | Yes (15) vs. No (92) | 1.585 | 0.908 – 2.768 | 0.105 |
| Extra-cerebral metastasis | Yes (76) vs. No (32) | 1.031 | 0.679 – 1.566 | 0.887 |
| **Hormone receptor positive** | **Yes (67) vs. No (38)** | **0.687** | **0.456 – 1.034** | **0.072** |
| **Endocrine therapy** | **Yes (53) vs. No (55)** | **0.704** | **0.477 – 1.038** | **0.076** |
| Trastuzumab | Yes (31) vs. No (77) | 0.808 | 0.525 – 1.242 | 0.331 |
| Triple negative^1^ | Yes (26) vs. No (78) | 1.255 | 0.800 – 1.968 | 0.323 |
| Bisphosphonate therapy | Yes (39) vs. No (69) | 0.978 | 0.652 – 1.465 | 0.913 |
| **Chemotherapy** | **Yes (94) vs. No (14)** | **1.685** | **0.914 – 3.107** | **0.094** |
| Dexamethasone therapy | Yes (91) vs. No (17) | 1.489 | 0.867 – 2.558 | 0.149 |
| RT technique^2^ | IMRT (48) vs. lateral opposing (49) | 0.976 | 0.649 – 1.466 | 0.906 |
| Radiotherapy boost ever | Yes (57) vs. No (51) | 0.834 | 0.566 – 1.228 | 0.358 |
| Radiotherapy boost, with one RT series only and w/o SRT^2^ | Yes (55) vs. No (42) | 0.734 | 0.484 – 1.115 | 0.147 |
| Subsequent RT series^3^ | Yes (5) vs. No (97) | 0.541 | 0.219 – 1.337 | 0.183 |
| **RT series almost complete^4^** | **Yes (94) vs. No (14)** | **0.153** | **0.079 – 0.295** | **2*10^-8^** |
| RT interruption | Yes (6) vs. No (102) | 1.622 | 0.703 – 3.745 | 0.257 |
| Global symptoms^5^ | Yes (28) vs. No (80) | 0.705 | 0.451 – 1.102 | 0.125 |

*Table 7: Univariable Cox regression analyses of variables tested for impact on the time interval between first diagnosis of cerebral metastasis and death.*

Variables included in the subsequent multivariable model are highlighted in bold.

^1^ In few cases, information is not available and thus sum of numbers is smaller than 108.

^2^ Without those six patients who underwent stereotactic radiotherapy techniques (SRT) and additional five who received more than one RT series.

^3^ Not considering the six patients irradiated with SRT.

^4^ At least 80% of WBRT or SRT total dose or WBRT ≥ 30 Gy.

^5^Global symptoms: Recurring headaches, nausea and vomiting, dizziness, fatigue, personality changes, confusion, and cognitive impairment
