# Supplementary material for: Borderline decisions in brain-metastatic breast cancer: efficacy of multimodality treatments in breast cancer patients with very limited prognosis suffering from brain metastases
Source: Clin Exp Metastasis. 2025 Oct 27;42(6):62. doi: 10.1007/s10585-025-10376-9 (PMC12559089; doi:10.1007/s10585-025-10376-9)
Supplement: Supplementary file 4 — Supplementary Material 4 [file 10585_2025_10376_MOESM4_ESM.docx]

| **Variables** | **Category level**  **(No. of patients)** | **Hazard ratio (HR)** | **95% CI for HR** | **P value (Cox)** |
| --- | --- | --- | --- | --- |
| RT series almost complete | Yes (94) vs. No (14) | 0.115 | 0.055 – 0.239 | 7*10^-9^ |
| Karnofsky Performance Status | ≤70% (70) vs. ≥80% (38) | 2.001 | 1.278 – 3.133 | 0.002 |
| Infratentorial metastasis | Yes (71) vs. No (37) | 1.792 | 1.146 – 2.804 | 0.011 |
| Chemotherapy | Yes (94) vs. No (14) | 2.067 | 1.080 – 3.958 | 0.028 |

Table 8: A multivariable Cox regression model was constructed using variables that showed univariable associations with p < 0.1, as highlighted in bold in Table 7. These eight variables, each represented by no more than one category level, were entered into a stepwise forward conditional Cox model. The results from the final iteration step are presented in this table.
